# Predictors of loss to follow-up among children in the first and second years of antiretroviral treatment in Johannesburg, South Africa

**DOI:** 10.3402/gha.v6i0.19248

**Published:** 2013-01-24

**Authors:** Mazvita Sengayi, Ntabozuko Dwane, Edmore Marinda, Nosisa Sipambo, Lee Fairlie, Harry Moultrie

**Affiliations:** 1 Wits Reproductive Health and HIV Research Institute, Faculty of Health Sciences, University of the Witwatersrand, Johannesburg, South Africa; 2 School of Public Health, Faculty of Health Sciences, University of the Witwatersrand, Johannesburg, South Africa

**Keywords:** HIV, antiretroviral treatment, children, loss to follow-up, South Africa

## Abstract

**Background:**

Ninety percent of the world's 2.1 million HIV-infected children live in sub-Saharan Africa, and 2.5% of South African children live with HIV. As HIV care and treatment programmes are scaled-up, a rise in loss to follow-up (LTFU) has been observed.

**Objective:**

The aim of the study was to determine the rate of LTFU in children receiving antiretroviral treatment (ART) and to identify baseline characteristics associated with LTFU in the first year of treatment. We also explored the effect of patient characteristics at 12 months treatment on LTFU in the second year.

**Methods:**

The study is an analysis of prospectively collected routine data of HIV-infected children at the Harriet Shezi Children's Clinic (HSCC) in Soweto, Johannesburg. Cox proportional hazards models were fitted to investigate associations between baseline characteristics and 12-month characteristics with LTFU in the first and second year on ART, respectively.

**Results:**

The cumulative probability of LTFU at 12 months was 7.3% (95% CI 7.1–8.8). In the first 12 months on ART, independent predictors of LTFU were age <1 year at initiation, recent year of ART start, mother as a primary caregiver, and being underweight (WAZ ≤ −2). Among children still on treatment at 1 year from ART initiation, characteristics that predicted LTFU within the second year were recent year of ART start, mother as a primary caregiver, being underweight (WAZ ≤ −2), and low CD4 cell percentage.

**Conclusions:**

There are similarities between the known predictors of death and the predictors of LTFU in the first and second years of ART. Knowing the vital status of children is important to determine LTFU. Although HIV-positive children cared for by their mothers appear to be at greater risk of becoming LTFU, further research is needed to explore the challenges faced by mothers and other caregivers and their impact on long-term HIV care. There is also a need to investigate the effects of differential access to ART between mothers and children and its impact on ART outcomes in children.

Sub-Saharan Africa is home to 90% of the world's 2.1 million HIV-infected children. Of all the people living with HIV in sub-Saharan Africa, 10% are children (1). In South Africa at the end of 2009, 330,000 children were infected with HIV and the prevalence of HIV in children aged between 2 and 14 years was 2.5% (1.9–3.5%) (2, 3). The number of patients receiving antiretroviral treatment (ART) at PEPFAR-supported government sites in South Africa increased nearly 20-fold in 5 years from 33,500 in 2005 to 632,000 in 2009 (4). In the same period, paediatric ART programmes in South Africa have seen an over 50-fold increase in average monthly patient enrolments from 2 to 106 (5). The impact of this rapid expansion of ART programmes on quality of care is a cause of concern.

The study of loss to follow-up (LTFU) and other treatment outcomes in HIV care has been used to monitor and improve programme effectiveness, using patient retention as a measure of quality of care (6). While children in HIV care programmes have a much higher retention than adults, a rise in LTFU has been observed as paediatric HIV care programmes scale-up (7–9). A trend of increasing LTFU over time was observed in a pooled analysis of the 16 paediatric HIV care programmes in sub-Saharan Africa which form the Kids’ Antiretroviral Treatment in Lower-Income Countries (KIDS-ART-LINC) Collaboration. The risk of LTFU was 2.8% (95% CI 1.9–4.1) at 6 months, 4.6% (95% CI 3.4–6.2) at 1 year, and 8.4% (95% CI 6.5–10.7) at 2 years (7).

Most children initiating ART in resource-poor settings start treatment at advanced stages of illness leading to high mortality rates especially in the first 3 months of ART initiation (1, 10–12). These early deaths if unreported may be misclassified as early LTFU. The risk factors of LTFU in the first year and second year of ART may differ. It is therefore important to examine the risk factors of LTFU in the first year on ART and explore whether these differ with predictors of LTFU after surviving the first year on ART. This is potentially useful in making recommendations for patient retention in paediatric HIV care programmes.

The aim of the present study was to determine the rate of LTFU in children receiving ART and to identify baseline characteristics associated with LTFU in the first year on treatment. We also explored the effect of patient characteristics at 12 months on LTFU in the second year on treatment.

## Methods

### Study population

The study is an analysis of prospectively collected routine data of HIV-infected children at the Harriet Shezi Children's Clinic (HSCC) in Soweto. The HSCC is a large urban paediatric HIV treatment clinic situated at the Chris Hani Baragwanath Academic Hospital, a referral hospital in Johannesburg. Since 1 April 2004, HSCC has treated children with government-funded ART. All children enrolled at HSCC are HIV-infected and ART is started based on current South African national guidelines. The 2004 national guidelines recommended ART for HIV-positive children with recurrent (two admissions per year) or prolonged (4 weeks) hospitalization, WHO clinical stage 3 and 4, or CD4 cell percentage <20% in children under 18 months and <15% for older children (13). The 2010 treatment guidelines recommend ART initiation for all HIV-positive children aged <1 year, for children aged 1–5 years with clinical stage 3 or 4 or CD4 ≤25% or absolute CD4 count <750 cells/mm^3^, and for children >5 years with clinical stage 3 or 4 or CD4 <350 cells/mm^3^ (14). Ethical approval for this study was granted by the University of Witwatersrand Committee for Research on Human Subjects.

All children <12 years of age who started ART at HSCC between 1 April 2004 and 30 October 2011 were included in the study. Twelve years was used as a cut-off because older children might have unique predictors of LTFU compared to younger children, and may be able to attend clinic visits on their own. Only children with a minimum follow-up time of 6 months before date of database closure were included in the study. The date of database closure was 30 April 2012. Children with follow-up time of <1 day (who never returned to the clinic after the day of initiation) were excluded from the analysis.

### Procedures

A child was defined as LTFU if their last date of contact with the clinic was >6 months before the date of database closure (30 April 2012), and they were not known to have died or transferred. Baseline exposure variables were as follows: age, sex, year of ART initiation; primary caregiver relationship; anthropometric measures (weight-for-age Z score (WAZ); height-for-age Z score (HAZ) and weight-for-height Z score (WHZ)); WHO clinical stage; CD4 cell percentage; immune suppression (as defined by the 2006 WHO classification of HIV-associated immunodeficiency in children using CD4 cell count and age) (15); log_10_ of plasma HIV viral load; and ARTregimen. Age was categorised into the following categories: <1 year, 1 to <3 years, 3 to <5 years, and 5 years to 12 years. Updated 12-month characteristics were used to investigate LTFU in the second year on ART. The United Nations General Assembly Special Session (UNGASS) on HIV/AIDS recommends reporting of 12-month outcomes of patients on ART and yearly thereafter as indicators of programme retention (16). This guided the selection of the 12-month cut-off in the analysis.

### Statistical analysis

Continuous variables were tested for the assumption of normality using histograms and normal quantile plots. Categorical variables were described using frequencies; normally distributed continuous variables in terms of mean and standard deviation; non-normal continuous variables in terms of median and inter-quartile range.

Time-to-event analysis was the primary method of analysis. In the analysis for LTFU in the first year on ART, person-time accrued from date of ART initiation to the earliest of (1) date of last visit, or (2) date at 12 months from ART initiation, or (3) date of database closure (30 April 2012). Cumulative probabilities of LTFU and period incidence rates were calculated. Kaplan–Meier curves were plotted and were compared using log rank tests. Cox proportional hazards models were fitted to investigate associations between baseline characteristics and LTFU. Global tests (using Schoenfeld and scaled Schoenfeld residuals) were used to test for validity of the proportional hazards assumption. Similarly, in the analysis of LTFU in the second year on ART, person-time accrued from 12 months post ART initiation to the earliest of (1) date of last visit, or (2) date at 24 months from ART initiation, or (3) date of database closure (30 April 2012). Variables included in the multivariate models were age, year of ART start, primary caregiver relationship, WAZ, and CD4 cell percentage. These variables were selected based on findings of other studies (5, 7, 8, 17), WHZ and HAZ were excluded for collinearity with WAZ, and regimen was excluded because the age at initiation determined regimen selection (13, 14).

Stata version 11 (Stata Corporation, College Station, Texas, USA) software package was used for all statistical analyses.

## Results

### Cohort description

**Fig. 1:**
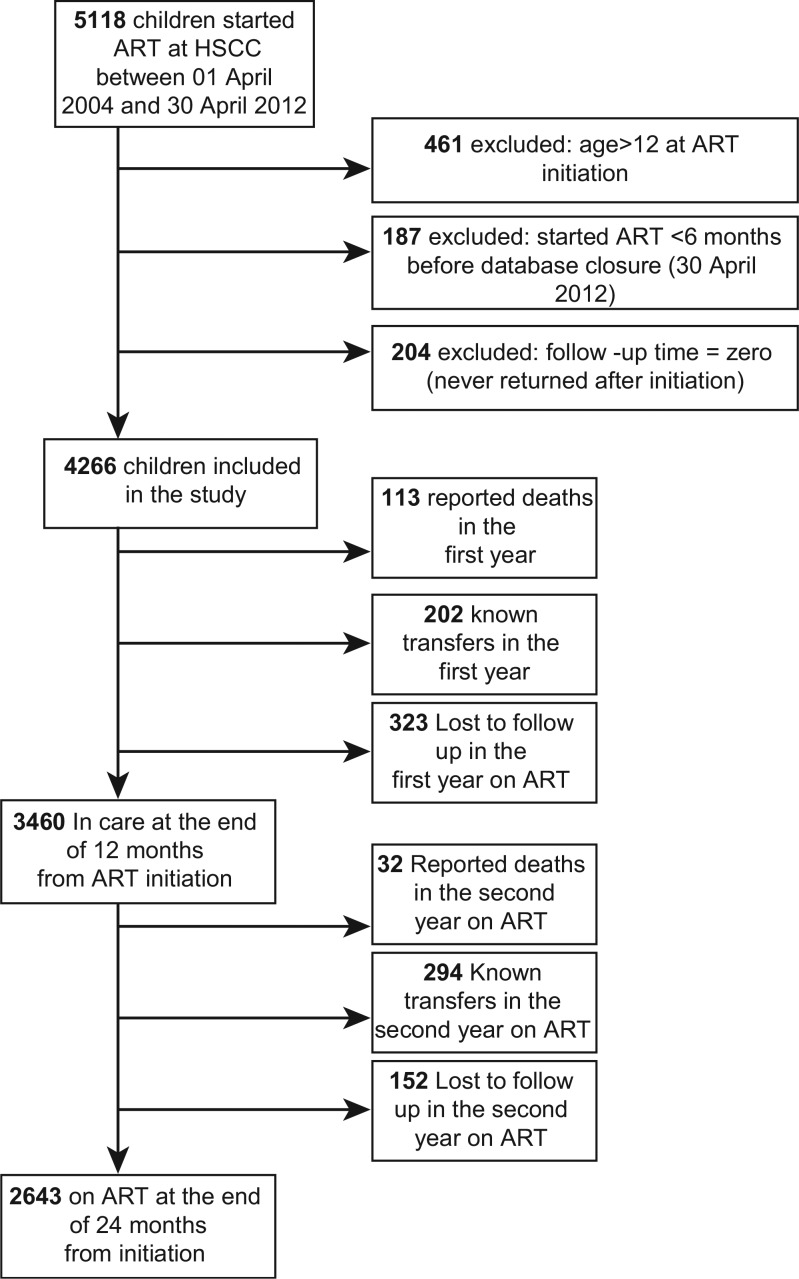
Flow chart of HIV-infected children initiating ART at HSCC between 01 April 2004 and 30 April 2012.

A total of 4,266 children enrolled between 1 April 2004 and 30 October 2011 were included in the study. A flow chart of children included in the study is shown in Fig. 1. Characteristics at baseline and at 12 months on treatment and the proportion of missing data for each variable are presented in Table 1. The median age was 4.2 years (IQR 1.4–7.4), and 48.7% (2078) of them were female. The majority of children (52.2%) had mothers as their primary caregivers at ART initiation. More than two-thirds of children had advanced/severe immunodeficiency (68.6%) at the start of treatment, and 73.7% had WHO clinical stage 3 or 4 disease. The mean CD4 cell percentage was 14.5% (SD 9.3) at baseline, and the mean log_10_ of HIV plasma viral load was 11.4 copies per millilitre (SD 2.6). Baseline regimens had either a protease inhibitor (PI) backbone (47.4%) or a non-nucleoside reverse transcriptase inhibitor (NNRTI) backbone (44.2%). At 12 months after starting treatment, 49.1% of the children in care were female and 52% had mothers as their caregivers. The proportion of children with advanced/severe immune suppression dropped to 48.1%, the mean log_10_ of plasma viral load dropped to 5.8 copies/mL (SD 3.6), and the mean CD4 cell percentage was 23.6 (SD 9.5).

**Table 1:** Overall cohort characteristics at baseline (*N*=4,266) and at 12 months on treatment (*N*=3,640)

	Baseline		12-month characteristics	
		
Characteristic	*N*	%	*N*	%
Sex				
Male	2188	51.3	1760	50.9
Female	2078	48.7	1700	49.1
Age				
< 1 year	823	19.3		
1 to <3 years	949	22.3	998	28.8
3 to <5 years	634	14.9	587	17.0
5 to 12 years	1860	43.6	1875	54.2
Median age, years (IQR)	4.2 (1.4–7.4)		5.2 (2.4–8.5)	
Year of starting ART				
2004–05	969	22.7	865	25.0
2006–08	1742	40.8	1548	44.7
2009–11	1555	36.5	1047	30.3
Primary caregiver				
Mother	2226	52.2	1800	52.0
Grandmother	641	15.0	594	17.2
Other family	827	19.4	741	21.4
Foster/institution/neighbour/guardian	301	7.1	256	7.4
Data missing	271	6.4	69	2.0
Weight-for-age Z score				
> −2 (not underweight)	3112	73.0	2430	70.2
−2 to −3 (moderately underweight)	461	10.8	543	15.7
< −3 (severely underweight)	369	8.7	426	12.3
Data missing	324	7.6	61	1.8
Mean WAZ (standard deviation)	−0.1 (2.6)		−0.8 (2.1)	
Height-for-age Z score				
> −2 (no stunting)	2911	68.2	2850	82.4
−2 to −3 (moderate stunting)	90	2.1	126	3.6
< −3 (severe stunting)	39	0.9	46	1.3
Data missing	1226	28.7	438	12.6
Mean HAZ (standard deviation)	2.7 (3.2)		1.9(2.8)	
Weight-for-height Z score				
> −2 (no wasting)	1273	29.8	1145	33.1
−2 to −3 (moderate wasting)	421	9.9	406	11.7
< −3 (severe wasting)	832	19.5	841	24.3
Data missing	1740	40.8	1068	30.9
Mean (standard deviation)	−2.2 (2.2)		−2.2(2.1)	
Immune suppression§				
Mild	263	6.2	1639	47.4
Advanced/severe	2927	68.6	1663	48.1
Data missing	1076	25.2	158	4.6
WHO clinical stage				
½	621	14.6	558	16.1
¾	3144	73.7	2815	81.4
Data missing	501	11.7	87	2.5
CD4 cell percentage				
≥ 15%	1327	31.11	2701	78.1
< 15%	1883	44.14	617	17.8
Data missing	1056	24.75	142	4.1
Mean (standard deviation)	14.5 (9.3)		23.6(9.5)	
Log_10_ of plasma viral load (copies/mL)				
Data missing	1158	27.1	177	5.1
Mean (standard deviation)	11.4 (2.6)		5.8 (3.6)	
Initial regimen				
NNRTI-based	1884	44.2	1251	36.2
PI-based	2024	47.4	1405	40.6
Data missing	350	8.4	804	23.2

§ Definitions of immune suppression according to the 2006 WHO classification of HIV-associated immunodeficiency in children by age and CD4%: (*children <1 year*: mild=CD4% of 30–35%, advanced=CD4% of 25–29%, severe=CD4% <25%; *children 1 to <3 years*: mild=CD4% of 25–30%, advanced=CD4% of 20–25%, severe=CD4% <20%; *children 3 to <5 years*: mild=CD4% of 20–25%, advanced=CD4% of 15–19%, severe=CD4% <15%; *children >5 years*: mild=CD4 cell count 350–499 cells/mm^3^, advanced=CD4 cell count 200–349 cells/mm^3^, severe=CD4 cell count <200 cells/mm^3^ or CD4% <15%) (14).

WHO: World Health Organization; NNRTI: non-nucleoside reverse transcriptase inhibitor (efavirenz or niverapine); PI: protease inhibitor (lopinavir/ritonavir).

### LTFU in the first year on ART

In the first year on ART, a total of 323 children were lost to follow-up (7.6%). There were a total of 3832.8 child-years of follow-up, and the overall incidence of LTFU in the first 12 months was 8.4 per 100 child-years (95% CI 7.6–9.4). The incidence of LTFU was highest in the first 3 months on ART with a period incidence rate of 13.6 per 100 child-years (95% CI 11.6–16.1). The cumulative probability of LTFU at 12 months was 7.3% (95% CI 7.1–8.8). There were 113 reported deaths and 202 known transfers in the first year (Fig. 1).

**Fig. 2:**
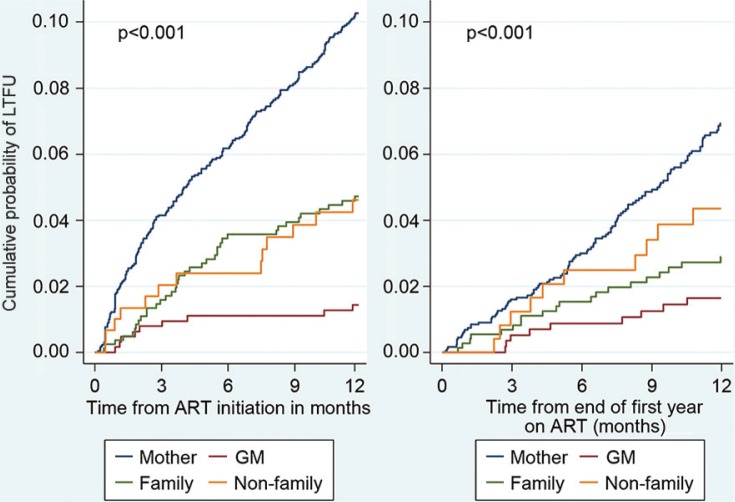
Kaplan–Meier estimates of cumulative probability of LTFU by caregiver relationship during the first year and second year on ART.

Age group, year of ART initiation, primary caregiver relationship, WAZ, and CD4 cell percentage were included in the multivariable Cox model to identify independent predictors of LTFU in the first year (Table 2). Older children were less likely to become LTFU than infants [HR 0.5 (95% CI 0.3–0.8) and HR 0.6 (0.4–0.9) for children aged 3 to <5 years and 5–12 years, respectively]. Children initiating ART in 2006–08 were twice as likely to become LTFU as those who initiated in 2004–05 [HR 2.1 (1.2–3.5)], and those initiating ART between 2009 and 2011 were five times more likely to become LTFU [HR 4.9 (2.9–8.2)]. Children whose biological mother was their primary care giver had the highest risk of LTFU. Having a grandmother [HR 0.1 (0.04–0.3)], other relatives [HR 0.6 (0.4–0.9)], or non-family caregivers [HR 0.4 (0.2–0.8)] as primary caregivers at baseline was significantly associated with a lower risk of LTFU than being cared for by the child's mother. Kaplan Meier plots also showed the increased risk of LTFU in children cared for by their mothers (Fig. 2). Severely underweight children (WAZ < −3) were over three times more likely to become LTFU than well-nourished children [HR 3.6 (2.5–5.3)]. CD4 cell percentage had no effect on risk of LTFU in the first year [HR 1.0 (0.99–1.0)].

**Table 2:** Baseline characteristics associated with LTFU in the first year on ART

Characteristic	Unadjusted HR (95% CI)	*p*	Adjusted HR* (95% CI)	*p*
Sex				
Male	1		–	
Female	0.94 (0.76–1.17)	0.580		
Age at ART initiation				
< 1 year	1		1	
1 to 3 years	0.79 (0.61–1.04)	<0.093	1.15 (0.79–1.69)	0.458
3 to <5 years	0.30 (0.19–0.45)	<0.001	0.47 (0.27–0.83)	0.009
5 to 12 years	0.31 (0.24–0.42)	<0.001	0.61 (0.41–0.93)	0.020
Year of starting ART				
2004–05	1		1	
2006–08	2.48 (1.56–3.93)	<0.001	2.05 (1.21–3.49)	0.008
2009–11	6.28 (4.05–9.75)	<0.001	4.90(2.92–8.20)	<0.001
Primary caregiver				
Mother	1		1	
Grandmother	0.13 (0.07–0.26)	<0.001	0.12 (0.04–0.31)	<0.001
Other family	0.45 (0.32–0.63)	<0.001	0.57 (0.37–0.88)	0.011
Foster/institution/neighbour/guardian	0.43 (0.25–0.76)	0.003	0.38 (0.18–0.82)	0.013
Weight-for-age Z score				
> −2 (not underweight)	1		1	
−2 to −3 (moderately underweight)	3.38 (2.52–4.53)	<0.001	2.71 (1.90–3.88)	<0.001
< −3 (severely underweight)	4.83 (3.61–6.49)	<0.001	3.64 (2.51–5.27)	<0.001
Height-for-age Z score				
> −2 (no stunting)	1		-	
−2 to −3 (moderate stunting)	11.10 (7.28–16.93)	<0.001		
< −3 (severe stunting)	15.17 (8.52–27.02)	<0.001		
Weight-for-height Z score				
> −2 (no wasting)	1		-	
−2 to −3 (moderate wasting)	0.89 (0.58–1.37)	0.592		
< −3 (severe wasting)	0.43 (0.28–0.67)	<0.001		
Immune suppression§				
Mild	1		-	
Advanced/severe	0.49 (0.33–0.71)	<0.001		
WHO clinical stage				
1/2	1		-	
3/4	1.37 (0.95–1.98)	0.090		
CD4 cell percentage	1.04 (1.02–1.05)	<0.001	1.01 (0.99–1.02)	0.212
Log_10_ of plasma viral load (copies/mL)	1.10 (1.04–1.16)	<0.001		
Initial regimen				
NNRTI-based	1		-	
PI-based	2.45 (1.88–3.21)	<0.001		

* *N*=3078 for adjusted model.

§ Definitions of immune suppression according to the 2006 WHO classification of HIV-associated immunodeficiency in children by age and CD4%: (*children <1 year*: mild=CD4% of 30–35%, advanced=CD4% of 25–29%, severe=CD4% <25%; *children 1 to <3 years*: mild=CD4% of 25–30%, advanced=CD4% of 20–25%, severe=CD4% <20%; *children 3 to <5 years*: mild=CD4% of 20–25%, advanced=CD4% of 15–19%, severe=CD4% <15%; *children >5 years*: mild=CD4 cell count 350–499 cells/mm^3^, advanced=CD4 cell count 200–349 cells/mm^3^, severe=CD4 cell count <200 cells/mm^3^ or CD4% <15%) (14).

WHO: World Health Organization; NNRTI: non-nucleoside reverse transcriptase inhibitor (efavirenz or niverapine); PI: protease inhibitor (lopinavir/ritonavir).

### LTFU in the second year on ART

At the end of 12 months from the start of ART, 3,460 children (81.1%) were still in care at HSCC. These children contributed a total of 3,064.4 child-years and the overall incidence of LTFU in the second year on ART was 5.0 per 100 child-years (95% CI 4.2–5.8). The cumulative probability of LTFU at the end of the second year was 4.9% (95% CI 4.2–5.7). There were 32 reported deaths and 294 known transfers in the second year (Fig. 1).

Table 3 shows adjusted and unadjusted hazard ratios for the effect of characteristics at 12 months treatment on LTFU in the second year. Age group at 12 months was not significantly associated with LTFU in the second year on ART. Children initiating ART in 2006–08 were twice as likely to become LTFU as those who initiated in 2004–05 [HR 1.9 (1.1–3.1)], and those initiating ART between 2009 and 2011 were nearly three times more likely to become LTFU [HR 2.7 (1.6–4.5)]. Children cared for by their grandmothers [HR 0.3 (0.1–0.6)] and other relatives [HR 0.6 (0.3–0.8)] had a lower risk of LTFU than those cared for by their biological mothers. Children who were still severely underweight after 1 year on ART were three times more likely to become LTFU than those who were well-nourished [HR 2.9 (1.9–4.5)]. The hazard of LTFU decreased by 3% for every unit increase in CD4 cell percentage [HR 0.97 (0.96–0.99)].

**Table 3:** The effect of 12-month characteristics on LTFU in the second year on ART

Characteristic	Unadjusted HR (95% CI)	*p*	Adjusted HR* (95% CI)	*p*
Sex				
Male	1		–	
Female	1.15 (0.83–1.8)	0.399		
Age at 12 months on ART				
1 to <3 years	1		1	
3 to <5 years	0.82 (0.53–1.29)	0.397	1.19 (0.74–1.91)	0.471
5 to 12 years	0.58 (0.41–0.83)	0.003	0.88 (0.58–1.35)	0.566
Year of starting ART				
2004–05	1		1	
2006–08	1.91 (1.19–3.08)	0.007	1.85 (1.12–3.07)	0.017
2009–11	2.84 (1.73–4.66)	<0.001	2.68 (1.58–4.55)	<0.001
Primary caregiver				
Mother	1		1	
Grandmother	0.23 (0.12–0.46)	<0.001	0.30 (0.15–0.59)	0.001
Other family	0.41 (0.26–0.67)	<0.001	0.49 (0.30–0.82)	0.006
Foster/institution/neighbour/guardian	0.63 (0.33–1.20)	0.155	0.59 (0.28–1.21)	0.150
Weight-for-age Z score				
> −2 (not underweight)	1		1	
−2 to −3 (moderately underweight)	2.09 (1.37–3.18)	0.001	1.97 (1.28–3.04)	0.004
< −3 (severely underweight)	3.51 (2.38–5.18)	<0.001	2.93 (1.91–4.47)	<0.001
Height-for-age Z score				
> −2 (no stunting)	1		–	
−2 to −3 (moderate stunting)	5.05 (2.60–9.81)	<0.001		
< −3 (severe stunting)	13.52 (6.19–29.55)	<0.001		
Weight-for-height Z score				
> −2 (no wasting)	1		–	
−2 to −3 (moderate wasting)	0.55 (0.28–1.09)	0.081		
< −3 (severe wasting)	0.64 (0.39–1.03)	0.068		
Immune suppression§				
Mild	1		–	
Advanced/Severe	1.74 (1.23–2.46)	0.002		
WHO clinical stage				
½	1		–	
¾	1.46 (0.88–2.43)	0.140		
CD4 cell percentage	0.98 (0.96–0.99)	0.007	0.97 (0.96–0.99)	0.004
Log_10_ of plasma viral load (copies/mL)	1.17 (1.12–1.21)	<0.001	–	
Regimen at 12 months				
NNRTI-based	1		–	
PI-based	1.43(0.93–2.20)	0.099		

* *N*=3,283 in adjusted model.

§ Definitions of immune suppression according to the WHO classification of HIV-associated immunodeficiency in children by age and CD4%: (*children <1 year*: mild=CD4% of 30–35%, advanced=CD4% of 25–29%, severe=CD4% <25%; *children 1 to <3 years*: mild=CD4% of 25–30%, advanced=CD4% of 20–25%, severe=CD4% <20%; *children 3 to <5 years*: mild=CD4% of 20–25%, advanced=CD4% of 15–19%, severe=CD4% <15%; *children >5 years*: mild=CD4 cell count 350–499 cells/mm^3^, advanced=CD4 cell count 200–349 cells/mm^3^, severe=CD4 cell count <200 cells/mm^3^ or CD4% <15%) (14).

WHO: World Health Organization; NNRTI: non-nucleoside reverse transcriptase inhibitor (efavirenz or niverapine); PI: protease inhibitor (lopinavir/ritonavir).

### Sensitivity analyses

Missing CD4 cell percentage values at baseline were imputed using the multivariate normal method, and the multivariate Cox model for the first year was re-run using the imputed values and compared with the multivariate Cox model using the original data, and similar results were obtained (Table 4). The multivariate Cox models were also re-run using age and year of start as continuous variables and compared with the Cox models with categorised age and year of start using likelihood ratio tests (Tables 4 and 5). There were no significant differences in models. Tests for linear trend confirmed a linear relationship between year of ART start and LTFU in the first and second year (Tables 4 and 5). Age group had a linear relationship with LTFU in the second year on ART (Table 5).

**Table 4:** Sensitivity analyses: LTFU in the first year

	Model A (original)		Model B (imputed CD4% missing values)		Model C (age and year of start as continuous)	
			
Characteristic	HR (95% CI)	*p*	HR (95% CI)	*p*	HR (95% CI)	*p*
Age at HAART initiation						
< 1 year	1		1			
1 to <3 years	1.15 (0.79–1.69)	0.458	0.89 (0.66–1.21)	0.452		
3 to <5 years	0.47 (0.27–0.83)	0.009	0.39 (0.24–0.63)	<0.001		
5 to 12 years	0.61 (0.41–0.93)	0.020	0.49 (0.35–0.69)	<0.001		
Age (as continuous variable)					0.93 (0.88–0.97)	0.002
Year of starting ART						
2004–05	1		1			
2006–08	2.05 (1.21–3.49)	0.008	2.08 (1.27–3.39)	0.003		
2009–11	4.90(2.92–8.20)	<0.001	4.67(2.90–7.50)	<0.001		
Year of starting ART (as a continuous variable)					1.36 (1.26–1.47)	<0.001
Primary caregiver						
Mother	1		1		1	
Grandmother	0.12 (0.04–0.31)	<0.001	0.18(0.09–0.37)	<0.001	0.12(0.04–0.31)	<0.001
Other family	0.57 (0.37–0.88)	0.011	0.64(0.45–0.93)	0.018	0.57(0.37–0.87)	0.010
Non-family	0.38 (0.18–0.82)	0.013	0.49(0.27–0.87)	0.015	0.40 (0.19–0.86)	0.019
Weight-for-age Z score						
> −2	1		1		1	
−2 to −3	2.71 (1.90–3.88)	<0.001	2.96(2.19–4.00)	<0.001	2.75 (1.92–3.93)	<0.001
< −3	3.64 (2.51–5.27)	<0.001	4.06(3.01–5.49)	<0.001	3.57 (2.48–5.18)	<0.001
CD4 cell percentage	1.01 (0.99–1.02)	0.212	1.01(0.99–1.02)	0.443	1.01 (0.99–1.02)	0.383

Likelihood ratio test between Model A and Model C showed that there was no difference between the model (*p*=0.449).

Tests for linear trend for age group were significant (*p*<0.001) and so were tests for departure from linear trend (*p*<0.001) suggesting a more complex relationship between age group and LTFU in the first year.

Tests for linear trend for year of ART initiation were significant (*p*<0.001) and those for departure from linear trend were not significant (*p*=0.935), hence there was a linear relationship between year of ART start and LTFU.

**Table 5:** Sensitivity analyses: LTFU in the second year

	Model D (original)		Model E (age and year of start as continuous)	
		
Characteristic	HR (95% CI)	*p*	HR (95% CI)*p*	*p*
Age at 12 months				
1 to <3 years	1			
3 to <5 years	1.19 (0.74–1.91)	0.471		
5 to 12 years	0.88 (0.58–1.35)	0.566		
Age (as continuous variable)			0.94 (0.89–0.99)	0.047
Year of Starting ART				
2004–2005	1			
2006–2008	1.85 (1.12–3.07)	0.017		
2009–2011	2.68 (1.58–4.55)	<0.001		
Year of starting ART (as a continuous variable)			1.21 (1.10–1.34)	<0.001
Primary caregiver				
Mother	1		1	
Grandmother	0.30 (0.15–0.59)	0.001	0.32(0.16–0.64)	0.001
Other family	0.49 (0.30–0.82)	0.006	0.54(0.32–0.89)	0.017
Foster/Institution/Neighbour/Guardian	0.59 (0.28–1.21)	0.150	0.63(0.30–1.29)	0.206
Weight-for-age Z score				
> −2 (Not underweight)	1		1	
−2 to −3 (Moderately underweight)	1.97 (1.28–3.04)	0.004	1.97 (1.29–3.04)	0.002
< −3 (Severely underweight)	2.93 (1.91–4.47)	<0.001	2.65(1.74–4.04)	<0.001
CD4 cell percentage	0.97 (0.96–0.99)	0.004	0.97 (0.95–0.99)	0.001

Likelihood ratio test between Model D and Model E showed that there was no difference between the model (*p*=1.000). No imputation of missing CD4% was done for the LTFU in the second year model since only 4.1% had missing CD4% at 12 months.

Tests for linear trend for age group at 12 months were significant (*p*=0.003) and those for departure from linear trend were not significant (*p*=0.525), hence there was a linear relationship between age group at 12 months and LTFU in the second year.

Tests for linear trend for year of ART initiation were significant (*p*<0.001) and those for departure from linear trend were not significant (*p*=0.559), hence there was a linear relationship between year of ART start and LTFU in the second year.

## Discussion

As in other paediatric HIV care programmes in sub-Saharan Africa (8, 10), the majority of children initiated ART at advanced stages of disease having WHO stage 3 or 4 and advanced or severe immune suppression. Incidence of LTFU in this study was 8.4 and 5.0 per 100 child-years in the first and second year of ART, respectively.

In the first year on ART the incidence rate of LTFU was highest in the first 3 months. This mirrors the previously reported high rate of death in the first 90 days on ART (18) and may be partly attributable to unreported early mortality. The cumulative probability of LTFU at 12 months was 7.3% (95% CI 7.1–8.8). This is comparable with the 1-year LTFU rate of 7% reported by the International epidemiologic Databases to Evaluate AIDS in Southern Africa (IeDEA-SA) study in a pooled analysis of 10 paediatric ART programmes from South Africa, Malawi, Mozambique, and Zimbabwe (10). The incidence of LTFU in Asian children on ART with an average follow-up time of 3 years was found to be lower at 4.2 per 100 person-years (19). In the first 12 months on ART, independent predictors of LTFU were age <1 year at initiation, recent year of ART, having one's biological mother as a primary caregiver, and being underweight (WAZ ≤ −2).The risk of LTFU increased progressively in successive enrolment calendar periods with those initiated between 2009 and 2011 having the highest LTFU. This increase in LTFU in children enrolled in more recent years is consistent with findings of studies of ART outcomes in similar settings (8, 17). This might reflect the effect of rapid scale-up and subsequently higher workloads on quality of care. A study of temporal trends in four South African provinces which comprised smaller, rural paediatric cohorts did not demonstrate the same progressive increase in LTFU in subsequent calendar cohorts as shown in this study (5). This suggests that the increase in LTFU might be related to rapid ART scale-up in large urban cohorts where the impact of high workloads on quality of care would be significant.

Haitian children who were LTFU had lower baseline median WAZ (−3, IQR −3.7 to −1.8) than those retained in care (20). WAZ is a marker of disease severity associated with mortality in the HSCC cohort (18). Among children who were still in care at 12 months since ART initiation, 12-month characteristics which predicted LTFU were recent year of ART, having one's biological mother as a primary caregiver, and being underweight (WAZ ≤ −2). The hazard of LTFU in the second year decreased by 3% for every unit increase in CD4 cell percentage. Baseline CD4 cell percentage had no effect on LTFU in the first year on ART, but children who still had a low CD4% after 12 months on ART were more likely to get LTFU in the second year. This may be explained by possible suboptimal adherence and consequent higher risk of death in children with a poor immune response at 12 months. Those who had higher CD4 cell percentage at 12 months were probably more adherent and less likely to suffer opportunistic diseases that may lead to death and LTFU.

The finding that children with their biological mothers as primary caregivers at baseline and at 1 year were more likely to be LTFU can be explained by the possibility that a number of these mothers may have died during the follow-up period resulting in the children becoming LTFU. Keeping HIV-positive mothers alive by effective ART has been shown to reduce under-five childhood mortality to levels seen in children of HIV-negative mothers (21). While it is likely that these mothers were enrolled in ART programmes themselves, it is possible that they might not be accessing ART due to the differences in the eligibility criteria for ART in adults and children in South Africa (13, 14, 22). At the time of the study, women would be initiated on ART if their CD4 count were ≤200 cells/mm^3^ or if they had a WHO stage 4 condition according to the 2004 guidelines (13). According to the 2010 ART guidelines women can access treatment at CD4 ≤200 cells/mm^3^ except in pregnancy and active tuberculosis, where therapy is started at CD4 ≤350 cells/mm^3^ (22). The paradox becomes that children are eligible for ART in South Africa at earlier disease stages than their non-pregnant mothers, with possible negative consequences on children's treatment outcomes. The adoption of the WHO Option B plus for prevention of mother to child transmission (PMTCT), which offers the best protection of maternal health by starting all pregnant HIV-infected women on ART for life (23), may positively impact children's outcomes.

Another explanation could be misclassification of mothers as caregivers since the caregiver status might not have been updated promptly on the HSCC database. A recent study of paediatric ART adherence in Gugulethu, Cape Town, showed that children cared for by their mothers were more adherent than those cared for by other relatives or foster parents, contradictory findings to those in our study (24).

This study draws its strength from a large sample size of children accessing care at the same site. The data was prospectively collected in an electronic format and includes social factors such as caregiver relationship which other cohorts elsewhere may not be able to collect adequately. Sensitivity analyses yielded similar results with the main analyses.

Our study had some limitations. The study was observational and key variables at baseline such as CD4 cell percentage, and log_10_ viral load had high proportions of missing data. Additionally, the quality of care at a referral academic hospital such the HSCC is likely to differ from that of lower levels of care or in rural areas. We used data from only one non-randomly selected urban site; therefore, these results cannot be generalised to children accessing care in non-urban settings.

## Conclusion

Increased roll-out of ART for HIV-infected children, particularly in recent years, has led to an increase in LTFU, especially in infants and strategies to retain infants and children in care need investigation. There are similarities between predictors of LTFU and known predictors of death. Unreported mortality possibly inflates LTFU in the first and second year of ART. Family-based care models improving maternal access to ART and reducing mortality need further exploration. The holistic care of HIV-positive children should emphasise linkage of caregivers to adult HIV care programmes. There is need to investigate the effect of differential access to ART between mothers and their children in South Africa and its impact on ART outcomes in children. There is need to strengthen ART roll-out programmes with resources to cope with rapid increases in enrolled patients.
